# Retraction Note: microRNA-193-3p attenuates myocardial injury of mice with sepsis via STAT3/HMGB1 axis

**DOI:** 10.1186/s12967-024-05807-2

**Published:** 2024-10-31

**Authors:** Jianyuan Pan, Buse Alexan, Dorn Dennis, Chiristine Bettina, Laeuf Ilona Mariya Christoph, Yongqin Tang

**Affiliations:** 1 Department of Cardiology, The First Affiliated Hospital of USTC, Division of Life Sciences and Medicine, University of Science and Technology of China, 230001 Anhui, China; 2 Institute of Experimental Cardiology, Internal Medicine VIII, Heidelberg University, Heidelberg, Germany; 3 Anatomy and Developmental Biology, European Center for Angioscience, Medical Faculty Mannheim, Heidelberg University, Heidelberg, Germany; 4 Department of General surgery, Chuzhou Hospital affiliated to Anhui Medical University, 230001 Anhui, China

**Retraction Note: Journal of Translational Medicine (2021) 19:386**

10.1186/s12967-021-03022-x

The Editor in Chief has retracted this article because its authorship could not be verified. The authors did not reply to requests for clarification and raw data. The Editor in Chief has, therefore, lost confidence in the integrity of the results reported in this article. The authors did not respond to correspondence from the Editor about this retraction.

